# Functional Dissection of Regulatory Models Using Gene Expression Data of Deletion Mutants

**DOI:** 10.1371/journal.pgen.1003757

**Published:** 2013-09-05

**Authors:** Jin'e Li, Yi Liu, Min Liu, Jing-Dong J. Han

**Affiliations:** 1 Center for Molecular Agrobiology, Institute of Genetics and Developmental Biology, Chinese Academy of Sciences, Chaoyang District, Beijing, China; 2 Graduate University of the Chinese Academy of Sciences, Beijing, China; 3 Key Laboratory of Computational Biology, Chinese Academy of Sciences-Max Planck Partner Institute for Computational Biology, Shanghai Institutes for Biological Sciences, Chinese Academy of Sciences, Shanghai, China; 4 Center of Molecular Systems Biology, Institute of Genetics and Developmental Biology, Chinese Academy of Sciences, Beijing, China; 5 Beijing Key Lab of Traffic Data Analysis and Mining, School of Computer and Information Technology, Beijing Jiaotong University, Beijing, China; University of Colorado, United States of America

## Abstract

Genome-wide gene expression profiles accumulate at an alarming rate, how to integrate these expression profiles generated by different laboratories to reverse engineer the cellular regulatory network has been a major challenge. To automatically infer gene regulatory pathways from genome-wide mRNA expression profiles before and after genetic perturbations, we introduced a new Bayesian network algorithm: Deletion Mutant Bayesian Network (DM_BN). We applied DM_BN to the expression profiles of 544 yeast single or double deletion mutants of transcription factors, chromatin remodeling machinery components, protein kinases and phosphatases in *S. cerevisiae*. The network inferred by this method identified causal regulatory and non-causal concurrent interactions among these regulators (genetically perturbed genes) that are strongly supported by the experimental evidence, and generated many new testable hypotheses. Compared to networks reconstructed by routine similarity measures or by alternative Bayesian network algorithms, the network inferred by DM_BN excels in both precision and recall. To facilitate its application in other systems, we packaged the algorithm into a user-friendly analysis tool that can be downloaded at http://www.picb.ac.cn/hanlab/DM_BN.html.

## Author Summary

The complex functions of a living cell are carried out through hierarchically organized regulatory pathways composed of complex interactions between regulators themselves and between regulators and their targets. Here we developed a Bayesian network inference algorithm, Deletion Mutant Bayesian Network (DM_BN) to reverse engineer the yeast regulatory network based on the hypothesis that components of the same protein complexes or the same regulatory pathways share common target genes. We used this approach to analyze expression profiles of 544 single or double deletion mutants of transcription factors, chromatin remodeling machinery components, protein kinases and phosphatases in *S. cerevisiae*. The Bayesian network inferred by this method identified causal regulatory relationships and non-causal concurrent interactions among these regulators in different cellular processes, strongly supported by the experimental evidence and generated many testable hypotheses. Compared to networks reconstructed by routine similarity measures or by alternative Bayesian network algorithms, the network inferred by DM_BN excels in both precision and recall. To facilitate its application in other systems, we packaged the algorithm into a user-friendly analysis tool that can be downloaded at http://www.picb.ac.cn/hanlab/DM_BN.html.

## Introduction

The complex functions in eukaryotic cells are implemented through a highly organized regulatory network composed of concerted activities of many genes and gene products. Gene expression can be directly regulated by transcription factors (TFs) [1], the states of chromatin structures [2], [3] and small RNAs, and interactions among them [4]–[6]. In other words, the mRNA expression level of a gene is the output synthesized from the information of several input signals.

Gene knockout is a classic approach to studying gene functions and the collection of yeast knockout strains has enabled systematic genome-wide functional analysis [7]. Transcriptional profiles of mutant strains have been used as molecular phenotypes for functional analysis and genetic epistasis analysis [8], [9]. In addition, the expression profiles of single, double and triple deletion mutants of chromatin machinery components, protein kinases and phosphatases were used to analyze the functional overlaps among these proteins [3], [10]. Dion *et al.* constructed 15 mutants of lysines 5, 8, 12, and 16 to arginine in the histone H4 tail and characterized the resulting genome-wide gene expression changes [11].

Transcriptional regulatory networks in different cellular contexts have been constructed through the DNA microarray analysis of transcription factor deletion mutants and over expression strains in *S. cerevisiae* [1], [12] by directly linking the genetically perturbed transcription factors (TFs) with the genes that change expression in response to the perturbations. As none of the regulators works alone, probably more important than constructing such regulator-target networks is to understand how the regulators cooperate to form regulatory pathways to specifically regulate a transcriptional program or biological processes [3].

Here we use the transcriptional profiles of deletion mutants as the molecular phenotypes of the mutants to determine how the regulators interact genetically or cooperate functionally with each other to modulate gene expression. We propose a Bayesian network (BN) approach to reverse engineer regulator networks from these gene expression profiles. The approach excels previous methods such as context-dependent regulation and correlation coefficient analysis [12]–[14] in that it can easily integrate different datasets and infer causalities in the regulatory program. Nodes in the network are the genes deleted in the mutants and the algorithm greedily searches over all possible Bayesian network structures for the one that best summarizes the relationships among the global differential expression change profiles upon deleting these genes. Thus by exploring the relationships among the global differential gene expression profiles for the deletion mutant genes, we can obtain valuable causal or non-causal relationships among these regulatory deletion-mutant genes through the inferred BN structure.

Then, we used the above approach to analyze the global differential gene expression profiles of 544 single or double deletion mutants of transcription factors, chromatin machinery components, protein kinases and phosphatases in *S. cerevisiae*. The BN inferred identified with high precision and recall causal regulatory and non-causal interaction relationships among these regulators in different cellular contexts.

## Results

### Pair-wise similarity between expression profiles of regulator mutants

The deletion mutants of transcriptional regulators used in this study are nonessential genes in yeast under rich medium growth conditions, yeast extract peptone dextrose medium (YPD) or synthetic complete medium (SC). We compiled expression profiles of sequence-specific DNA binding transcription factors (STFs) deletion strains grown in SC and YPD mediums [12], [15]. We also collected the expression profiles for deletion mutants of protein kinases, phosphatases [10] and chromatin machinery components [3] (See Methods for more detailed data descriptions).

To confirm that regulators belonging to the same protein complex or regulatory pathway tend to share common targets [15], we used Jaccard similarity index (JI) to examine the similarities between targets profiles of the perturbed regulators (see Methods, Figure S1A, Table S1) and we observed that a STF is more likely to connect with another STF than with a general transcription regulator (GTFs e.g. chromatin modifiers and remodelers) whether the regulators are derived from the same data set or different data sets. Indeed, the percentage of known physical interactions or genetic interactions (downloaded form SGD) present among predicted gene pairs increases as the threshold of pair-wise Jaccard similarity index (*JI*) used in prediction increases (Figure S1B), suggesting that the similarities of gene expression profile changes after genetic perturbation of transcriptional regulators can be used to infer relationships among these regulators. However, *JI* is only a crude measure that is subject to different cutoffs and cannot infer directionality or causality of regulatory relationships. In contrast, Bayesian network is a solid statistical inference method that can infer directions or causality of regulatory relationships and is more appropriate for this task.

### A perturbation based Bayesian network structure learning algorithm

A Bayesian network [16] is a directed probabilistic graphical model which represents conditional independency relationships between variables. The BN learning approach has been extensively used in previous works to analyze gene expression and other high throughput data sets [14], [17], [18]. Suppose that the expression of a deletion mutant gene (denoted by G) is fully determined by its three intermediate regulator genes (denoted by A, B, C), if the expression of genes A, B, C can be controlled precisely, we can find a specific expression configuration of A, B, C (e.g., A is up-regulated and B, C are down-regulated) so that the expression of G is as small as possible just like being deleted. As such, we can anticipate that the global differential gene expression profile of deleting G versus the wild type strain can be well predicted from the global differential expression profiles of deleting B, deleting C and over-expressing A, respectively. Although the datasets contain only genetic deletion strains, no over-expression strains, the global differential expression profile of the profile of over-expressing A is often opposite to that of deleting A, we can thus well predict the differential gene expression pattern of deleting G from the three differential gene expression profiles of deleting genes A, B and C, respectively. In general, if one gene is combinatorially regulated by a set of other genes, usually we can approximate its deletion-mutant differential expression ‘phenotype’ fairly well by the deletion-mutant differential expression ‘phenotypes’ of its regulator genes.

However, in deletion mutant experiments, it is typical that most genes have small expression changes in deletion mutant strains compared to their WT. For instance, 80% yeast genes have similar expressions to the WT strain in protein kinase or phosphatases deletions under the same growth condition [19]. Thus, the differential expression profiles of these regulators are sparse. The majority of ‘neutral’ gene expression changes (represented by ‘0's) in the differential expression profiles will artificially induce a high similarity between the deletion mutant genes (regulators) in classic BN learning methods.

**Figure 1:**
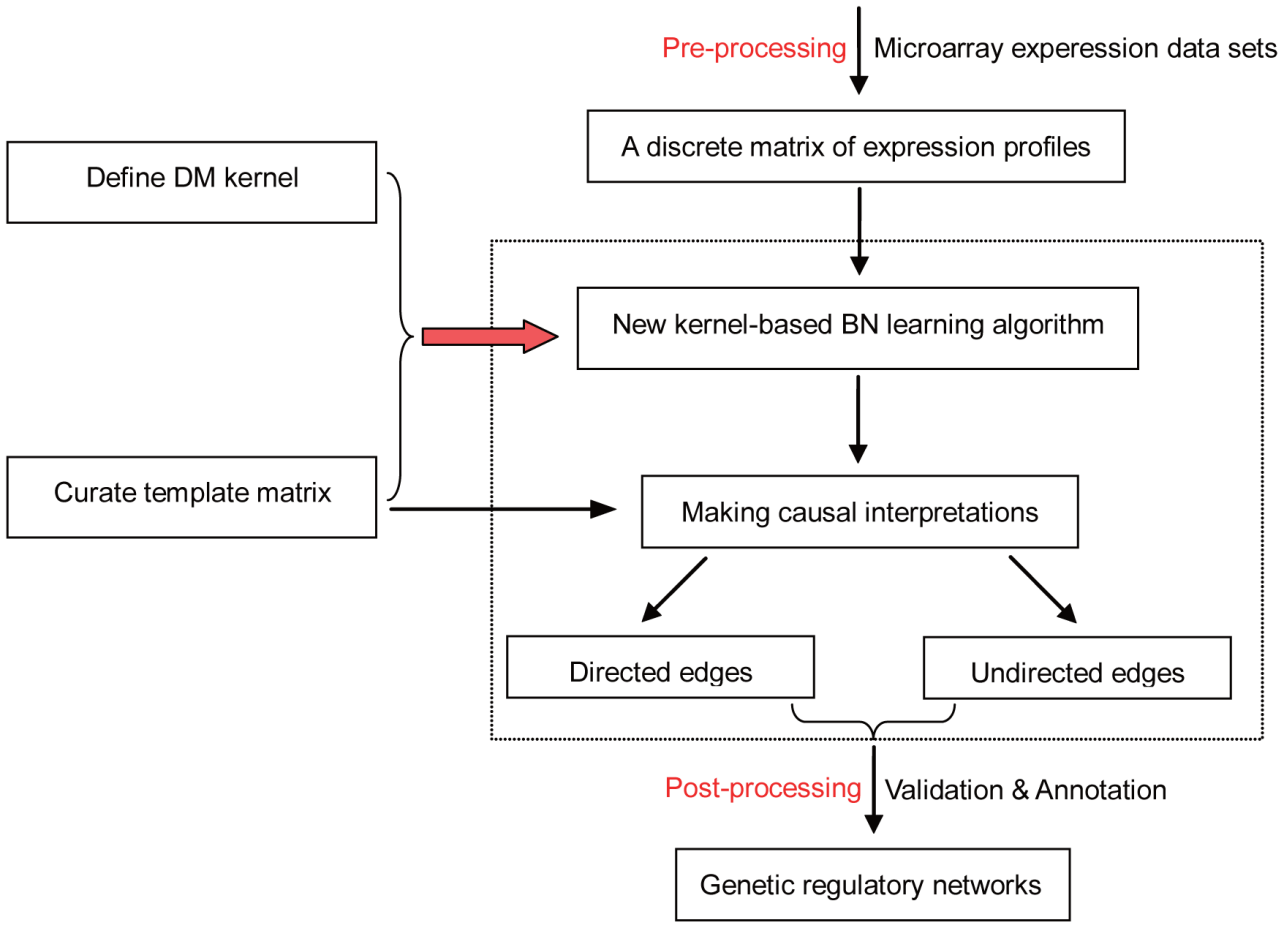
Overview of the Bayesian network learning algorithm DM_BN for reverse engineering regulatory networks from genetic perturbation data. As an example, DM_BN takes as input deletion mutant gene expression profiles. The relative change of the mRNA expression levels of the deletion mutant strains versus the wild type (WT) is represented by 1 (significant up-regulation), −1 (significant down-regulation) and 0 (no significant change). The algorithm incorporate a new kernel to model the consistent gene expression changes upon perturbation and it employs a template of all potential regulator-regulator interactions to enable more accurate and much faster BN learning. After learning the BN structure, Meek's rule [28] is used to infer compelled (directed) and non-compelled (undirected) edges. The precision of the inferred network is then assessed by existing knowledge of protein complexes, regulatory relationships (MAPK signaling transduction pathways) and gene annotations.

To this end, we developed a new Bayesian network structure-learning algorithm called Deletion Mutant BN (DM_BN) (Figure 1), which is specifically designed for reverse engineering regulatory networks of deletion mutant genes from differential gene expression profiles in the corresponding deletion mutant strains. Note that, the input of this algorithm is a matrix of discrete values: 1, −1, 0, which denote the differential gene expression of the mutant strain versus the WT. Each column of the matrix records the differential gene expression profile for one deletion mutant gene. As described above, the training data for Bayesian network is skewed towards 0, it is not viable to exploit classical Bayesian network learning approaches based on discrete data [20]. Indeed, in our extensive comparison of the proposed DM_BN algorithm with state-of-the-art BN learning algorithms with three other scoring metrics [20]–[23], a well-known software package for BN learning [24] and two widely used non-Bayesian approaches to building regulatory networks [25], [26] on the yeast deletion mutant datasets, the significantly improved network inference quality fully confirmed the advantage of the DM_BN algorithm (See below).

The main technical contribution of the DM_BN algorithm is to employ the kernel based approach to Bayesian network inference [27] and the introduction of a novel kernel for discrete data that is specifically designed for characterizing the deletion mutant data sets. Specifically, suppose 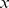 and 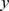 are two discrete variables which could take values 1, −1, 0, the trivial kernel for discrete data in [27] is defined as: 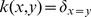, i.e., 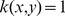 when 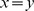; and 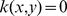 when 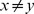. This is not viable for dealing with the deletion mutant data sets since the dominant value in such data is 0, the trivial kernel for discrete data will induce a large similarity output (1.0) for all most all gene pairs which are neither up-regulated nor down-regulated. To prevent this biased effects, we modified the trivial kernel to the DM kernel below:
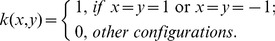
The implication from the new DM kernel is clear: the differential expression changes of two genes in a deletion mutant experiment are considered similar (with kernel output 1.0) if they are either up-regulated or down-regulated simultaneously. The similarity between genes that are not responsive to the deletion mutant experiment is abandoned (with kernel output 0.0). In this way, only the information of the co-regulation activity is fed into the Bayesian network-learning algorithm (Methods).

Another contribution of the DM_BN algorithm is the incorporation of the *a priori* knowledge from deletion mutant experiments into Bayesian network learning. For this purpose, we employ a network template to constrain the space of graph search in Bayesian network learning and to provide additional causal information in the learning and interpretation of Bayesian network structure. The basic idea of constructing the adjacency matrix of the network template (template matrix for short) is as follows: First, we start with an empty template matrix of zeros. Then, we define the list of target genes of a deletion mutant gene to be the genes whose mRNA levels either up- or down-regulated compared to the WT strain. If both deletion mutant genes *A* and *B* (with indices 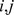, respectively) are not in the target gene list of each other, the two genes do not seems to have a direct regulatory relationship, but they could cooperate to regulate other genes. So, if the target gene list of *A* and *B* overlap (i.e., at least one gene appear in both of the two target gene lists), the 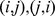 elements of the template matrix are set to 1, which means that either one of the two edges 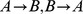 might appear in the final BN. Finally, if gene *B* appears in the target gene list of *A*, but *A* is not in the target gene list of *B*, we set 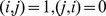, which means that 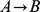 could appear in the final BN while the reversed edge 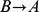 is forbidden. In rare occasions, when both A, B appear in the target gene list of each other, we set 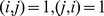, since we do not know which direction of the interaction represents the dominant regulatory effect while the other represents the secondary feedback effect (Figure 1).

To identify potential causal interactions from Bayesian network structure, we have to determine whether the directionality of each edge in the network is reversible or not [28]. In this step, the template matrix again provides *a priori* causal information to guide the algorithm to disambiguate more edge directionalities. More details of the algorithm are presented in Methods.

### Performance evaluation of BN structure inferences

To quantitatively compare the performance of the DM_BN learning algorithm with other approaches to infer regulatory networks, we curate a database of ground-truths protein-protein interactions, regulatory interactions, genetic/epistatic interactions and protein complexes from the KEGG and SGD databases. Here, methods being compared include alternative Bayesian network learning algorithms (the WinMine Toolkit [24], the BDeu scoring approach [22] with optimized prior [23] and the BIC scoring approach [20], [21] and non-Bayesian network approaches (the ARACNE [25] software, the Disruption Network [26] and the Jaccard similarity index (*JI*) approach). Details of these algorithms and the strategies used in the testing are described in Methods and Supplemental Note 1 (Text S1).

**Figure 2:**
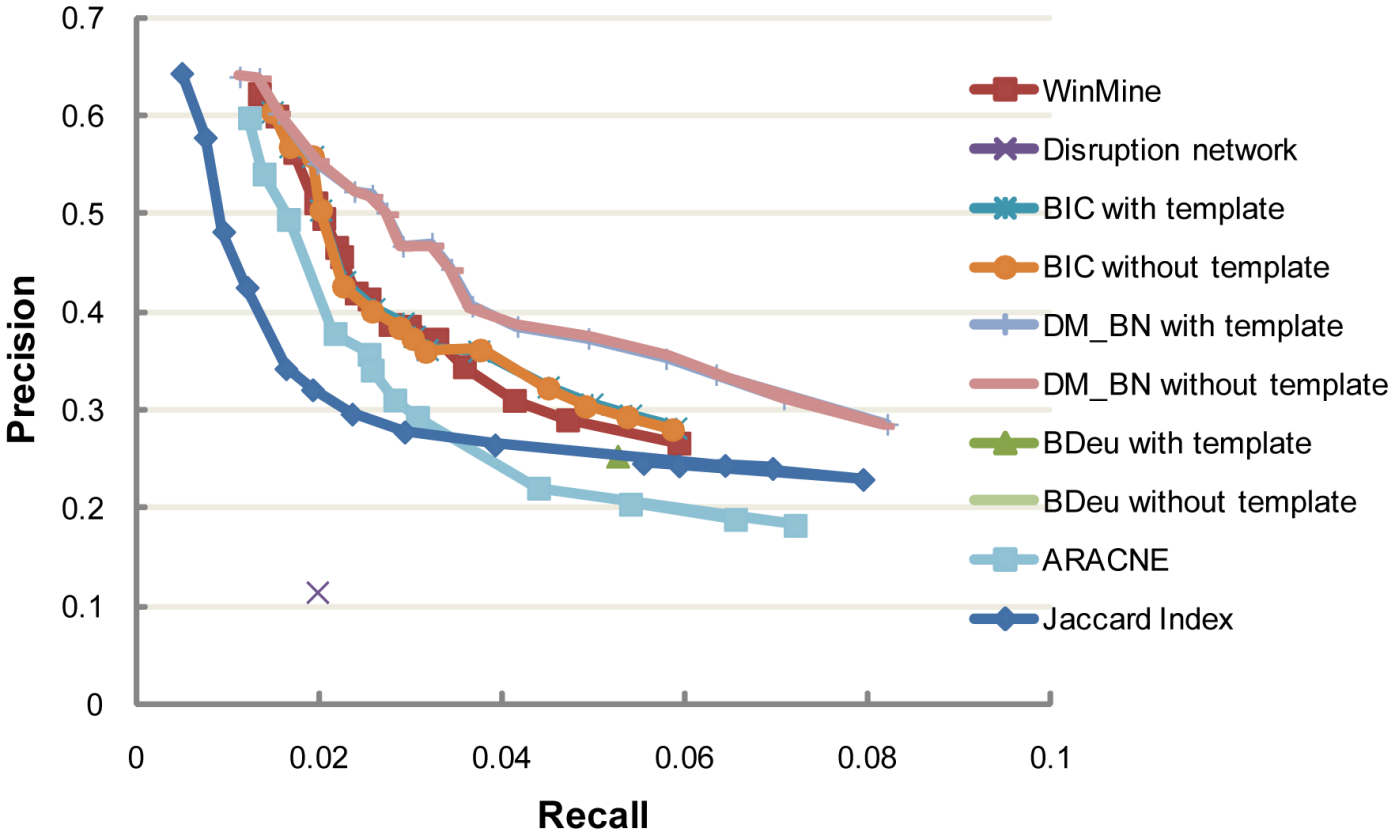
Precision and recall of networks inferred by DM_BN and by other network inference methods at various cutoffs. The colored lines show the precision and recall tradeoffs as a function of different values of Jaccard index threshold, mutual information threshold in ARACNE, kappa parameter in WinMine, weight of the penalty term in BIC and the 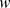 parameter in DM_BN, respectively. The Disruption network approach and the BDeu metric do not have any tunable parameter and only one network can be predicted. We curated 5662 protein-protein interactions, regulatory interactions, genetic/epistatic interactions and protein complexes from the KEGG and SGD databases to evaluation the performance of different algorithms (See Table S2).

Basically, two key performance indicators are important for comparing the above algorithms: 1) Precision-recall curve, which quantifies the ability of an algorithm to correctly predict *bona fide* interactions between these regulators; 2) The precision of orientation, which measures the ability of an algorithm to predict correct directionality for each causal interaction. We first calculated the precision and recalls of all the predicted yeast regulator networks (Methods). In this computation, directionality is not considered in matching a predicted edge and a known interaction in the database, which is partly because we only have very limited knowledge about the causality of these ground-truth interactions. By plotting the corresponding precision-recall points (or point, if an algorithm predicts only one network) for each algorithm, we found that DM_BN algorithm outperforms all the alternative network construction approaches in both precision and recall (Figure 2, Table S2). In other words, regardless of causality, DM_BN algorithm has the highest precision of *de novo* network predictions over the whole range of recall rates.

**Figure 3:**
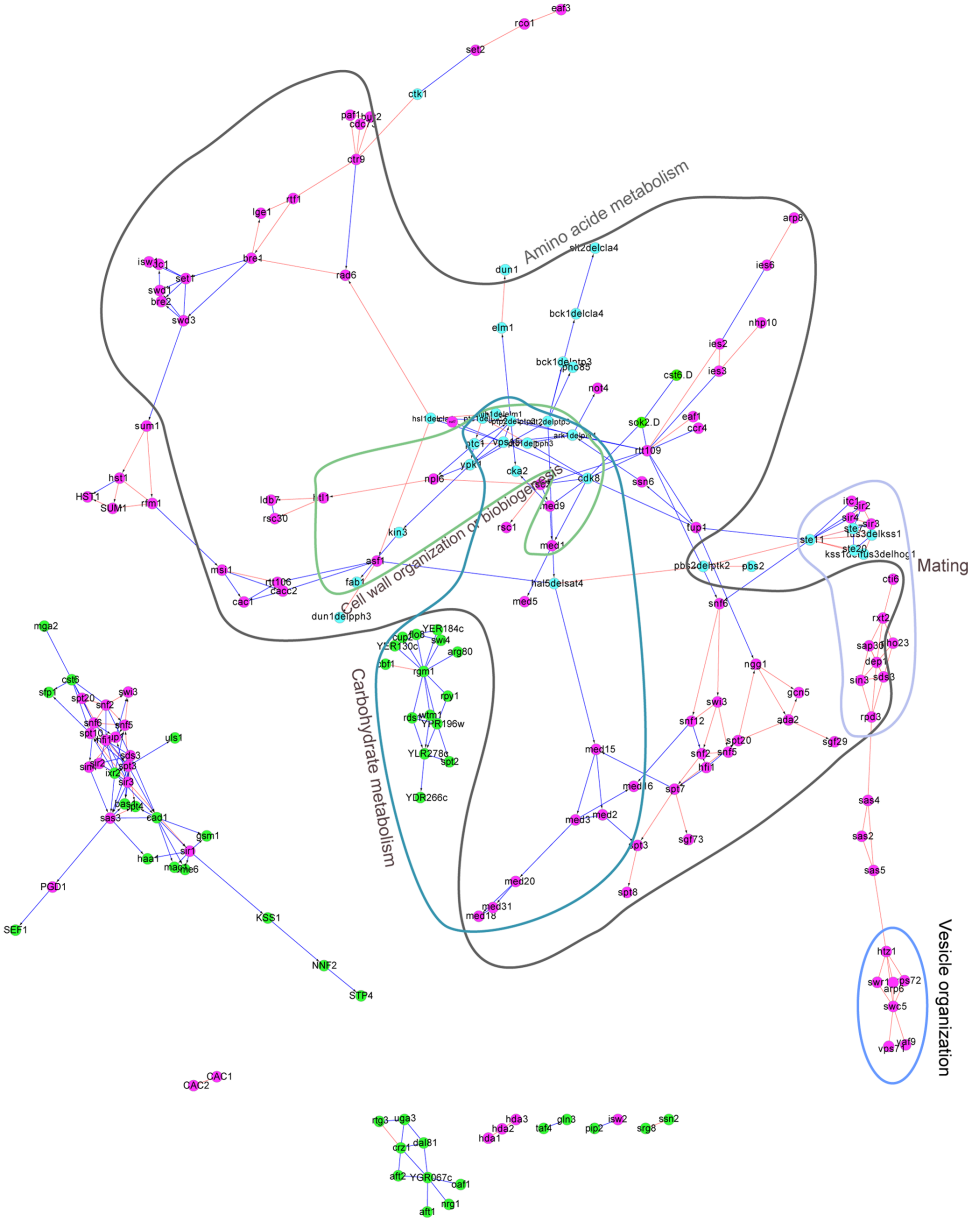
The yeast regulatory network inferred from the deletion mutant gene expression profiles by DM_BN. Each node in this network represents a single or doubly mutated regulator(s). The directionality of edges indicates predicted causal relationship between the regulators. Known regulator interactions (physical or epistatic interactions) were shown by red colored edges. Regulators involved in the same cellular process, such as amino acid metabolism, cell wall organization or biogenesis, are highlighted by circles with annotations. Purple nodes: chromatin machinery components (mostly general transcription factors); Cyan nodes: single or double mutants of protein kinases or phosphatases; Green nodes: sequence-specific DNA binding transcription factors. The parameter in the DM_BN algorithm was set to 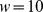.

A close examination of the BN inferred by the DM_BN algorithm suggested it indeed recapitulated many interactions in protein complexes or pathways. Specifically, the BN structure visualized in Figure 3 with precision 0.4704 and recall rate 0.0323 (Figure 2) includes both causal (represented by directed edges) and non-causal (by undirected edges) relationships among these regulators, which are known to take place in diverse biological processes to combinatorially regulate the expression of target genes. Moreover, we also computed the functional enrichment of these regulators based on their target genes (Methods). The result suggests that regulators that tightly interconnected in the BN more significantly share common functions than other regulator pairs (Figure 3, Table S3). The network learned by DM_BN algorithm further predicts how these regulators interact with each other in different cellular processes (Figure 3).

For example, the predicted network module among subunits of the chromatin remodeling machinery complex (Figure 3, shown by purple nodes) has a high precision of 0.85217 (Table S4); the network module consisting of protein kinases Vps15, Ark1, Prk1, Cdk8, Cka2 and protein phosphatase Ptc1, Ptc2, Pph3, Ptp3 is involved in three interrelated cell processes: cell wall organization or biogenesis, amino acid metabolism and carbohydrate metabolism, which is consistent with biological knowledge; and the predicted network suggests that Rpd3 complex, Sir complex and Ste11 mediated MAPK kinase cascades pathway cooperate with each other in mating process (Figure 3, Table S3). We also observed that a STF is more likely to connect with another STF than with a GTF, which is similarly observed in the densely connected network inferred by the Jaccard index (*JI*) similarity measure (Figure S1A). Our results are also consistent with the E-MAP results, which are quantitative genetic interactions between phosphorylation related genes in *S. cerevisiae* [19]. For instance, it is known that histone variant H2A.Z (encoded by the Htz1 gene) exchange with histone H2A in nucleosomes through the SWR1 complex [29], [30] and that Htz1 displays positive genetic interactions with SWR1 (+3.5), Vps71 (+3.9) and Vps72 (+3.5) [19]. These interactions are all predicted by the network inferred by DM_BN (Figure 3). Indeed, the target sets of Htz1, SWR1, Vps71 and Vps72 deletion mutants have high similarity, with Jaccard indices (Methods) 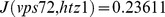 
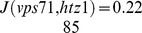
 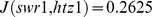 (Figure 3 (blue circle), Table S5). Moreover, the functional enrichment of the predicted SWR1 complex target gene sets for vesicle organization (Table S3) is consistent with the fact that SWR1 complex is required for vacuolar protein sorting [31]. More examples of the inferred pathway relationships are listed in Table S6, S7.

### Prediction of causal relationships

Inferring correct directionalities for causal interactions or epistasis is an important aspect for regulatory network predictions. However, most non-Bayesian network algorithms are unable to do so. For example, the ARACNE [25] software and the Jaccard similarity index (*JI*) approach could only predict undirected interactions. Although the Disruption Network [26] could predict the direct causal relationships between deletion mutant genes and differentially expressed genes, such knowledge is derived from the deletion mutant experiments without performing causality inference. It is of special interest to see if an algorithm can make *de novo* predictions about causality among deletion mutant genes from the similarity of their genome-wide differential expression profiles. In principle, Bayesian network learning algorithms hold this promise and thus we compare the performance of the four BN learning algorithms (DM_BN, Winmine toolkit [24], the BDeu [22] and BIC scoring approaches [20], [21]) in predicting causal relationships.

Since the ground-truth causal relationships derived from existing databases for the 378 regulator genes is very limited, and also because we do not know the exact cellular contexts in which those causal relationships hold true, to quantify the performance in predicting causal relationships, we calculated the recall and the precision of all these network inference approaches except BDeu using the four MAPK (mitogen-activated protein kinase) cascades where clear causal relationships are well described among these kinases. The exclusion of BDeu here is simply because it does not have a tunable parameter to generate a relative sparse network that is comparable to the size of networks generated by the other three approaches. However, using a different evaluation approach, BDeu's causality prediction apparently does not perform as well as DM_BN and BIC (see below). Yeast contains at least four MAPK (mitogen-activated protein kinase) cascades that convert extracellular stimuli into intracellular signals during a variety of cellular processes, such as mating, cell wall remodeling and high osmolarity adaption [32]. We found that when all tools predicted roughly the same number of edges, the DM_BN algorithm with prior information can predict more interactions with correct orientations than other tools among kinases involved in the same signal transduction pathway (Table S8, S9, S10, S11).

To test whether the correct inference of edge direction is solely the result of applying a template, we examined the directionality of edges in BNs inferred by DM_BN without any template. At various parameters, 73.3–89.2% of the edges have the same direction as the regulator-DEG relationships identified in the deletion mutants experiments. These proportions are significantly higher than that expected by chance (random coin tossing p = 0.5, Binominal test p = 0.0625∼5.42e-07, Table 1). This indicates that the correct inference of edge directions by DM_BN is largely not attributed to using the template. However, the BN inferred by DM_BN with the template, did correct a small number edges incorrectly predicted when not using the template (1/34∼4/15 edges, Table 1). This is because the network template not only corrects edge orientation errors inconsistent, but also improves the global causal structure in the BN through the cascading interactions between edges. Therefore, a template is included in the actual implementation of the DM_BN algorithm as the default setting.

**Table 1:** Precision of edge orientations for networks inferred by DM_BN with/without template at different 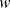 parameters.

*ω* of DM_BN		1.5	2	2.5	3	4	5,6,8,9	7
No. of regulator- DEG relationships predicted by DM_BN without template	No. of total edges	37	29	20	15	11	8	7
	No. of edges with correct direction	33	24	16	11	9	7	6
	Precision	0.8919	0.8276	0.8000	0.7333	0.8182	0.8750	0.8571
Binomial test (p≥0.5)	P-value	5.42e-07	0.0003	0.0059	0.0592	0.0327	0.0352	0.0625
No. of regulator- DEG relationships predicted by DM_BN with template	No. of total edges	34	25	19	15	11	9	8
	No. of edges with correct direction	34	25	19	15	11	9	8
	Fraction corrected	1/34	1/25	3/19	4/15	2/11	2/9	2/8

Using the regulator-DEGs identified in the deletion mutants experiments as reference, the “No. of total edges” represents the total number of predicted causal interactions in a BN that overlap with the reference regardless of edge direction. The “No. of edges with correct direction” represents the number of predicted causal interactions with the same orientation as the reference. The “Fraction corrected” represents the percentage of edge orientations corrected by using the template in the DM_BN algorithm.

Using a similar approach, we also compared with other BN inference algorithms, the performance of DM_BN in *de novo* predicting causal relationships without using the *a priori* information encoded by the template. The DM_BN algorithm and the BIC scoring approach [20], [21] generally predict non-compelled directed edges remarkably more precise than the BDeu scoring method [22] or the WinMine toolkit [24] (Supplemental Note 2 and 3 in Text S1, Figure S2).

**Figure 4:**
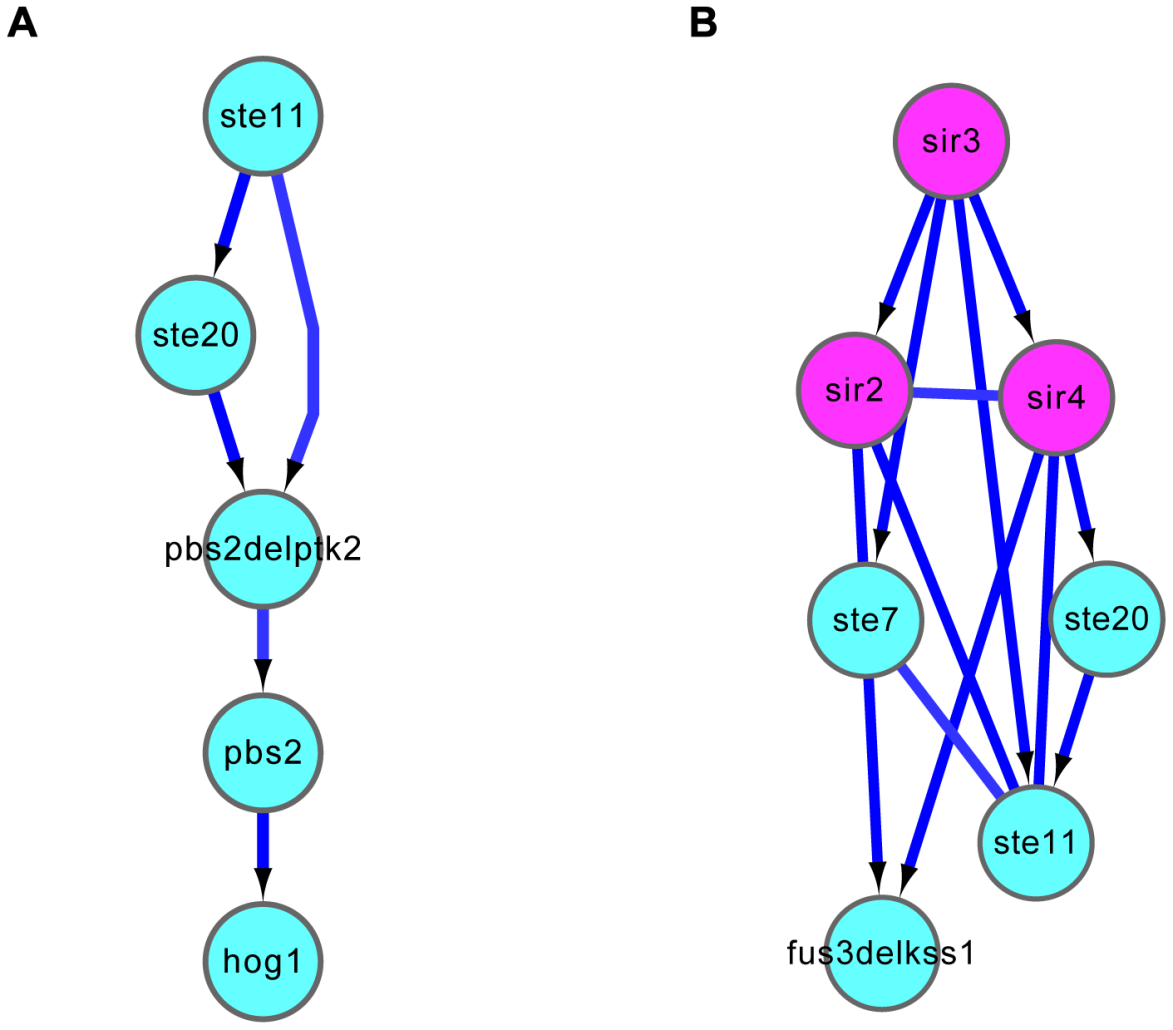
Causal relationships predicted by DM_BN. (A) Linear cascading relationships among regulators involved in HOG signaling pathway in response to osmotic stress were correctly inferred. (B) The predicted relationships between the SIR complex and the STE/FUS3/KSS MAPK signal transduction pathway are consistent with their roles in mating process. Purple nodes: components of the SIR complex; Cyan nodes: single or double mutants of protein kinases. The parameter in the DM_BN algorithm for generating these predictions was set to 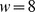. See section “Novel regulator-regulator interactions predicted by DM_BN” for more details.

In particular, the causal relationships inferred by the DM_BN algorithm (with the network template) correctly recapitulated the linear cascade structure for regulators in the HOG signaling pathway involved in the osmotic stress response (Figure 4A). For instance, Ste11 MAPK kinase (MAPKKK) phosphorylates Pbs2 MAPK kinase (MAPKK). Then, the activated Pbs2 phosphorylates Hog1 in the MAPK kinase cascade pathway for osmostress adaptation [33].

In the mating process, DM_BN not only accurately grouped the SIR complex and the Ste11 mediated MAPK cascade pathways, but also correctly predicted the connectivity among components of the complex or the pathway (Figure 4B). The results correctly recovered the role of Ste7 and Ste11 protein kinases in two different MAPK Fus3 and Kss1 cascade pathways that controls mating, respectively [33], [34].

### Novel regulator-regulator interactions predicted by DM_BN

The inferred causal relationships or non-causal interactions between these gene expression regulators not only confirmed known relationships, such as physical interactions and genetic epistasis relationships among these regulators, but also predicted many novel relationships that could be important in gene regulation.

**Figure 5:**
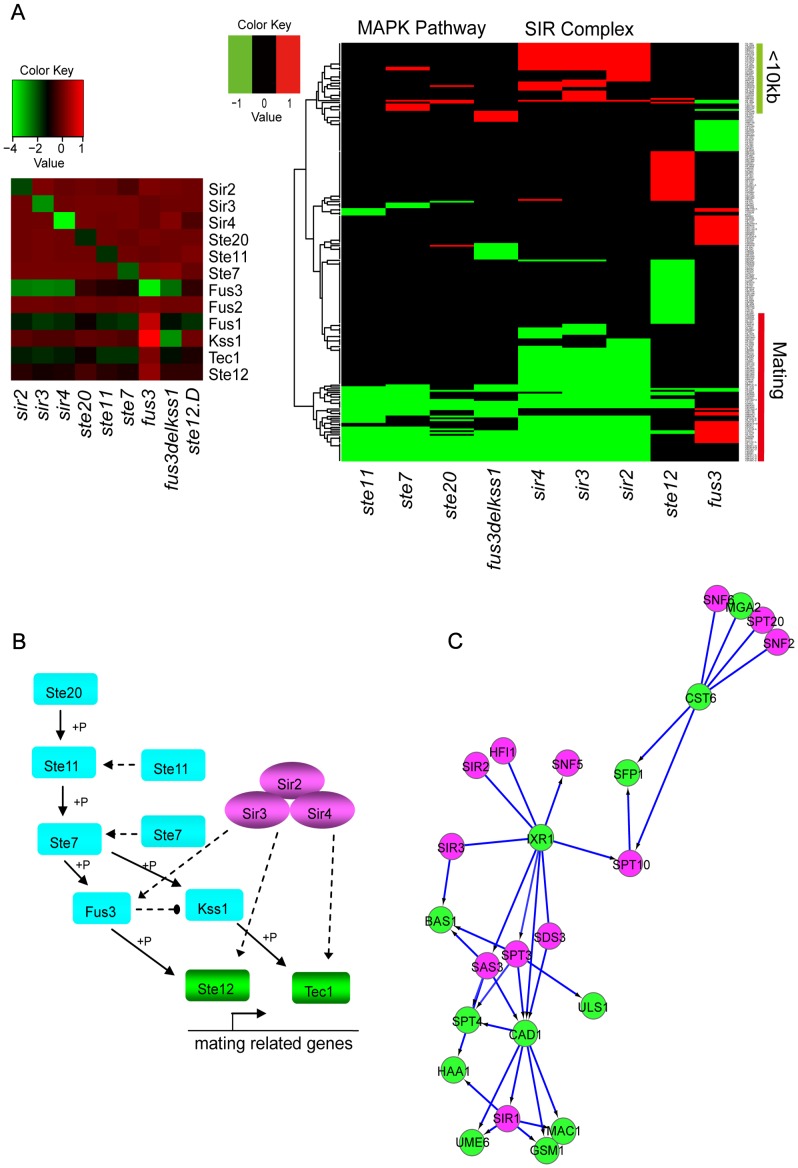
Novel causal or non-causal relationships predicted by DM_BN and an inferred novel regulatory model involved in mating process. (A) Left panel: All genes that are significantly changed compared to the wild type upon deletion of subunits of the Sir Complex and kinases of Ste11-mediated MAPK cascading pathway. Right panel: Genes marked by green bar are <10 kb from the nearest telomeres, and genes marked by red bar are involved in mating process. (B) A novel predicted regulatory network that SIR complex and (Ste11-mediated) MAPK cascades pathway has functional overlap through kinase Fus3 (that were down regulated in the SIR complex) in mating. Known Ste11-mediated MAPK pathway is shown by Solid lines. Solid lines: known interactions, dashed lines: newly predicted interactions. (C) Novel causal relationships and undirected interactions predicted by DM_BN among STFs: Cst6, Sfp1, Bas1, Mac1, Gsm1, Ixr1, Haa1, Ume6 and Cad1, and some subunits of the SIR, SWI/SNF and SAGA complex, under parameter setting 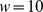. Purple nodes: chromatin machinery components; Green nodes: sequence-specific DNA binding transcription factors.

For example, the DM_BN algorithm not only correctly predicted the connection between components in the SIR complex or in the MAPK pathway, but also predicted the dense connection between the SIR complex and Ste11-mediated MAPK cascades (Figure 4B, Table S3 and Table S12). Clustering of the expression profiles of the genes in these network modules shows that the genes up-regulated in the deletion mutants of Sir2, Sir3 and Sir4 (Figure 5A, right panel) are all within 10 kb to their nearest telomere. Meanwhile, the predicted functions of the genes down-regulated by all deletion mutants in Figure 5A are enriched for mating process (see also Table S3). All these findings are consistent with the knowledge that SIR complex plays roles in silencing at HML, HMR loci which carry unexpressed copies of mating-type genes and telomeres [35] and that SIR complex is comprised of two structural proteins Sir3 and Sir4, deletion of which will cause reduced mating rate at different levels [36]. The mRNA expression levels of Fus3 and Fus1 are very low in the deletion mutants of Sir2, Sir3 and Sir4 in all the three data sets (Figure 5A, left panel). However, the mRNA levels of other genes in the model are not changed compared to WT (except the expression levels of the deletion mutant genes themselves) (Figure 5A, left panel). From the causal, non-causal relationships predicted by DM_BN (Figure 4B) and the expression profiles of deletion mutants experiments (Figure 5A), we can infer a novel model implying that the Ste11 mediated MAPK cascades pathway may have overlapping functions with the SIR complex (Figure 5B). Thus, SIR complex could indirectly influence the mRNA expression of kinase Fus3, which is involved in the MAPK cascades pathway in mating process.

Although a STF is more likely to have a similar gene expression pattern with another STF than with a GTF generally (Figure 3A), the network suggests that STFs Cst6, Sfp1, Bas1, Mac1, Gsm1, Ixr1, haa1, Ume6 and Cad1 connect densely to GTFs (some subunits of SIR complex, SWI/SNF complex and SAGA complex) (Figure 5C). Clusters (Figure S3) of the expression profiles of these regulators revealed high similarities between the target profiles of the STFs, Sfp1 and Cst6, and the GTFs SWI/SNF complex and SAGA complex. Another example is the high similarity between the targets profiles of STFs: Ixr1, Cad1, Bas1 and Stp4; and GTFs: SIR complex, SAGA complex. Although no physical interactions or binding relationships between them have been reported in the literature, SAGA subunit Spt3 has been reported to have negative genetic interactions with Stp4 and Ixr1 [10]. These novel predictions by the DM_BN algorithm may serve as blueprints for further experimental explorations.

## Discussion

Uncovering complex regulatory networks is an important and challenging task [1], [12], [19], [37]. Here, we introduced a new Bayesian network inference algorithm “DM_BN”, specifically designed to infer regulatory networks from gene expression profiles generated by gene perturbations, such as gene deletions. DM_BN can work with both small and large datasets and infer causal and non-causal relationships among the perturbed genes. To address the sparsity of gene expression changes in the perturbation experiments, we developed a kernel-based BN learning algorithm DM_BN, which is appropriate for modeling such gene expression data sets. Comparing with known biological interactions, both the recall and the precision of the network inferred by the proposed DM_BN algorithm are significantly higher than that inferred by WinMine and by the Jaccard Index (*JI*) similarity measure (Figure 2). The DM_BN network model not only successfully recapitulated known interactions among the yeast transcriptional regulators, but also predicted many novel interactions among these regulators and regulatory protein complexes, offering new insights into the yeast transcriptional regulatory network.

Our results show that the improved performance of the DM_BN algorithm can be mainly ascribed to the new kernel. Since an edge between two regulator genes is allowed in the network template if they share at least one target gene, the template matrix actually allowed all the possible interactions between these genes, hence has a very little predictive value by itself. However, the DM_BN algorithm still benefits from using the network template in two aspects. First, by eliminating all impossible edges, the template effectively reduced the search space to speed up the DM_BN algorithm. Second, by encoding the *a priori* regulator-target causal knowledge in deletion mutant experiments, the network template not only corrects edge orientations that are inconsistent with such information (Table 1), but also improves the global causal structure predicted by DM_BN through edge-edge interactions, as we demonstrated in the inference of MAPK pathways (Table S11).

Although the DM_BN approach has achieved big success in inferring yeast regulatory network from perturbation-based gene expression data sets, there are still a few limitations to its applications. For example, the mRNA expression levels of target genes are not fully representative of the activities and interactions of the regulators in modulating gene expression. This is because post-transcriptional changes and the regulators' context-specific transient activity were not measured in the experiments. Due to the intrinsic limitation of mRNA expression data, our method failed to identify certain relationships among the regulators under certain conditions, especially when the activity of the regulators is not screened in the microarray experiments. Nevertheless, these problems are not the fault of the proposed BN inference algorithm but rather inherent limitations of current experimental systems, which are expected to overcome by introducing other types of high-throughput datasets. In this sense, the application of the DM_BN algorithm is not limited to microarray expression profiles of genetic perturbations, it can actually be extended to work on many kinds of high-throughput data, such as epigenomic, transciptomic, proteomic data sets, and even quantitative phenotype data.

## Methods

### Data sets

All the gene expression profiles are downloaded from the Gene Expression Omnibus (GEO) database, including 269 transcription factors knockout strains grown in yeast extract peptone dextrose medium (YPD) [12], 150 deletion mutants of protein kinases and phosphatases [10], 165 mutants of chromatin machinery components [3] and 52 sequence-specific DNA binding transcription factors (STFs) deletion strains grown in synthetic complete medium (SC) [15]. Altogether the four data sets above contain gene expression profiles of 544 yeast deletion mutants. The series accession numbers of these data sets are GSE4654 [12], GSE25644 [10], GSE25909 [3] and GSE2324 [15].

### Gene expression data analysis

The detailed DNA microarray normalization and statistical analysis procedures see described in Supplemental Methods (Text S1). After processing, the gene expression changes are represented by discrete values: 1 (significant up-regulation), −1 (significant down-regulation) and 0 (no significant expression change).

### Inference of the optimal Bayesian network structure

We employ the kernel-based Bayesian network learning algorithm [27] with three modifications. First, we use the ‘DM kernel’ instead of the trivial kernel to handle the yeast deletion mutant datasets. Second, we use a template matrix to constrain the space of all possible Bayesian network structures. Details of the ‘DM kernel’ and the construction of the template matrix are described in the Results and will not be repeated here. Finally, we modified the BIC scoring function by increasing the weight of the complexity term for penalizing the Kernel Generalized Variance [38] measure. This is necessary for removing biological noise and increasing the precision and sparsity of the finally obtained network structure. Formally, the Bayesian network scoring function is modified as follows (cf. eqn. 4 in ref [27] for details):
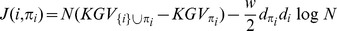
Here, 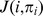 is the BIC score for node 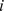 and its parents 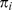, and the overall score 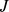 for a full Bayesian network is: 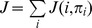. 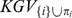 and 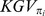 are the Kernel Generalized Variance [38] for node sets 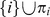 and 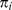. 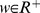 is the multiplicative weight that we impose on the second term of the scoring function.

With the DM kernel inside the KGV measure, the template matrix as a structural constraint and the modified scoring function, we can search for the Bayesian network structure that optimally fit the yeast deletion mutant datasets. Specifically, in each step, we consider 1) adding an edge that is consistent with the template; 2) deleting an edge from the current BN structure; 3) reversing the direction of an edge that will not violate the causal constraints embodied the template. In accordance with previous studies, we use the greedy ascent TABU search method [39] to find the ideal Bayesian network structure. Here, ‘TABU’ denotes a Meta searching strategy that prohibits the algorithm from ‘undoing’ a recent operation. It helps the search procedure from being getting stuck in the local optima regions [39]. Finally, we adopt an efficient dynamic graph acyclicity checking method [40] in the Bayesian network structure search, since the most computational intensive task in this study involves inferring a Bayesian network of up to ∼400 nodes, using the conventional static graph acyclicity checking method would be fairly slow.

### Deriving causal knowledge from the Bayesian network structure

Interpreting the causalities in the Bayesian network structure is not a straight forward task. This is because there are equivalence classes of Bayesian network structures. All BNs in the equivalence class are semantically equivalent. They share the same set of skeletons (edge connections regardless of arrows), but differ in the directionalities of some edges [41]. As such, there are two types of edges in a Bayesian network: compelled edges, whose directionalities are fixed among BNs in the equivalence class and non-compelled edges, whose directionalities are not consistent in the equivalence class [42]. The authors also proposed an efficient algorithm to dissect a Bayesian network into compelled (directed) edges and non-compelled (undirected) edges (the results are collectively represented as a partially directed acyclic graph, a.k.a., PDAG) [42]. However, the approach is not well suited for our study because the template matrix contains much *a priori* causal information, which was not used by Chickering's algorithm [42].

To overcome this problem, we employ Meek's rules [28] to convert the Bayesian network structure into a PDAG. The merit of this algorithm is that prior causal knowledge could be fully exploited in making causal interpretations of the Bayesian network structure. As such, we extract asymmetries in the template matrix and impose those constraints in the causal interpretation algorithm [28]. In this way, the causal information conveyed in deletion mutant experiments is used maximally.

### Jaccard index

Jaccard's similarity index [43] quantifies the similarity between two sets of elements. In this work, we use Jaccard index to evaluate the similarity between the deletion mutant expression profiles of two regulators, which is calculated as follows:
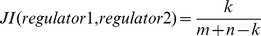
Here:


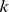
: No. of common targets for regulator1 and regulator2.


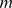
: No. of genes whose expression significantly changed in the deletion mutant of regulator 1.


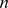
: No. of genes whose expression significantly changed in the deletion mutant of regulator 2.

### Evaluating the Precision/Recall of the inferred network

We use known relationships between the 165 mutants in 30 chromatin modification complexes [3], ground-truths protein-protein interactions, regulatory interactions and genetic/epistatic interactions curated from the KEGG and SGD databases to evaluate the precision/recall for the predicted networks. For this purpose, we calculated the recall and the precision of a network using the formula below:
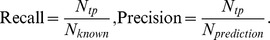


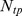
: the number of edges that are correctly predicted in the network (true positives, i.e., predicted edges that are also consistent with known protein-protein interactions).


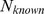
: the total number of known pair-wise interactions between nodes in the network (e.g., between subunits in known protein complexes).


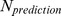
: the number of edges in a predicted network.

In rare cases, a deletion-mutant regulator gene appears in more than one data set. Removing any copy of that gene will typically cause many of its interactors in that data set undetected. So, multiple copies of all these overlapping genes are retained in our analysis. In this scenario, no matter how many edges between two same genes from two different datasets are predicted in the network, we only count this interaction once. Moreover, in the analysis of causal relationships, if the directionalities of these edges are consistent, we retain the directionality of that interaction; otherwise, we treat this interaction as an undirected (non-compelled) edge.

### Functional enrichment analysis

The significance of the functional enrichment of a gene list is computed by performing the hypergeometric test. In this work, all the GO annotation, phenotype and pathway data sets were downloaded from the SGD, KEGG database. The *P*-value is calculated as follows:
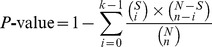


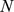
: The total number of genes with significant expression change in any deletion mutant experiment.


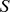
: Number of genes in one particular functional category.


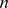
: Number of genes with significant expression change in the current deletion mutant experiment.


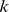
: Number of overlapped genes between the *n* genes and the *S* genes.

### Strategies for systematic testing and evaluation of network-inference methods

To systematically compare the performance of the DM_BN algorithm with existing network-inference methods, we tested the ARACNE [25] software, the Disruption Network approach [26] and the Jaccard Index (*JI*) approach, which are not based on Bayesian networks. We also tested the WinMine Toolkit [24] and Bayesian network learning with two other scoring metrics (BIC [20], [21] and BDeu [22]) (Detailed description of each method is listed in Supplemental Note 1 in Text S1).

To ensure the comprehensiveness of the evaluation, for each method and whenever possible, we use a wide range of parameter settings to infer a number of networks to best reveal the tradeoffs between precision and recall. Specifically, for the DM_BN algorithm, we run it with a wide range of values for the 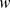 parameter (See Methods “Inference of the optimal Bayesian network structure” for details) to infer a set of BNs with different number of edges. Similarly, for the WinMine toolkit [24], we run it at different values of the kappa parameter to infer BNs; for ARACNE [25], various mutual information thresholds are used to infer a set of regulatory networks; for the Jaccard index (*JI*) approach, a wide range of the *JI* similarity cutoffs are used for network construction and for the BIC score approach, different weights (like the 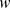 parameter in DM_BN) are multiplied to the penalty term (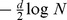, see Eqn. 42 in [20]) in the Bayesian information criterion to infer BNs. Note that we could only generate a single best network for the BDeu scoring approach [22] using the optimal ESS value [23] since there is no way to tune the precision-recall tradeoffs. Similarly, only a single network can be inferred for the disruption network approach [26]. This is because to determine whether a gene is significantly differentially expressed in a deletion mutant strain, we employed the default statistical tests and significance thresholds used in the original experimental study (See “Statistical analysis of expression profiles” in Supplemental Methods). It is not clear how to adjust the thresholds synchronously for the four data sets.

## Supporting Information

Figure S1Jaccard's similarity between the target gene sets of regulators. (A) Dense pair-wise similarities between the gene expression profiles of deletion mutants. Edges connecting two nodes were drawn in the figure if the Jaccard similarity index between their targeting gene sets is higher than a specific threshold 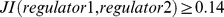. A STF (Green nodes) is more likely to connect to another STF than to a GTF (Purple nodes). (B) Left panel: the similarity level of deletion mutant expression profiles (quantified by the thresholds of Jaccard's index used in the prediction) is correlated with the likelihood of being known interactions (protein-protein interactions and epistatic relationships downloaded from SGD). Right panel: The number of predicted and known interactions that passed the thresholds of Jaccard's similarity index. KI: known protein-protein interaction and epistatic relationships. PI: predicted protein-protein interaction and epistatic relationships. KI%: the percentage of known protein-protein interactions or epistatic relationships among gene pairs that passed the thresholds of Jaccard's similarity index. Purple nodes: chromatin machinery components (GTFs); Cyan nodes: single or double mutants of protein kinases, phosphatases; Green nodes: sequence-specific DNA binding transcription factors (STF).(PDF)

Figure S2Precision of edge orientations for networks inferred by DM_BN, BIC, WinMine and BDeu at different parameters. BNs are predicted by four template-free BN learning algorithms (as indicated in the key) at various parameter settings. Using the regulator-DEGs identified in the deletion mutants experiments as reference, the x-axis represents the total number of predicted causal interactions in a BN (i.e., the directed edges in the corresponding PDAG) that overlap with the reference regardless of edge direction. The y-axis represents the precision of edge orientation (the percentage of predicted causal interactions with the same orientation as the reference). Tunable parameters include the *kappa* parameter in WinMine, the weight of the penalty term in BIC and the 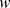 parameter in DM_BN. The BDeu metric does not have any tunable parameter and only predicts one network. Note that the recall of a BN prediction cannot be calculated based on these reference relationships, because simply linking regulators to DEGs identified from the deletion mutants experiments are too permissive to represent the direct regulator-target gene relationships and hence cannot be used as a gold standard. DEG: Differentially expressed genes.(PDF)

Figure S3 Clustering of the deletion mutant expression profiles of the STFs and GTFs shown in Figure 5C.
(PDF)

Table S1The pair-wise Jaccard similarity index (JI) values between target gene sets for deletion mutants of the regulators shown in Figure S1. 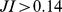.(XLS)

Table S2 Known interactions downloaded from the SGD data set. —— undirected interactions; ——> directed interactions.
(XLSX)

Table S3 Function enriched among a regulator's target genes under rich medium growth conditions.
(PDF)

Table S4Evaluation of the WinMine and DM_BN algorithm based on known knowledge of chromatin modification complex. For a fair comparison, parameters for the two algorithms were set to 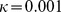 in WinMine and 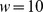 in DM_BN, so that the networks inferred by different methods have roughly the same number of edges.(DOCX)

Table S5 Similarity between the target gene sets of subunits of the SWR1 complex and Htz1, which are involved in vesicle organization. See Figure 3 for their predicted relationships by DM_BN.
(DOCX)

Table S6The predicted interactions by WinMine when 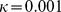. —— undirected interactions; ——> directed interactions. Networks shown in Table S6 and S7 were used for functional enrichment analysis and for predicting the chromatin modification complex relationships.(XLSX)

Table S7The predicted interactions by DM_BN with network template when 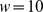.(XLSX)

Table S8The predicted interactions by WinMine when 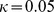.(XLSX)

Table S9The predicted interactions by DM_BN with network template when 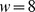.(XLSX)

Table S10The predicted interactions by DM_BN without network template when 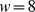.(XLSX)

Table S11Evaluating the causality predicting performance of WinMine, BIC and DM_BN with/without the network template using known knowledge of the MAPK signaling pathways. The relationships were predicted by WinMine under the parameter setting 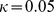, by BIC under the penalty term's weight 2.0 and by DM_BN under 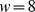.(PDF)

Table S12 Similarity between target gene sets of subunits in the SIR complex and kinases in the STE-mediated MAPK signaling pathways. In this table, we list the Jaccard index similarities between regulators involved in mating and filamentous growth processes. See Figure 3 for more information for their predicted relationships.
(DOCX)

Text S1 Supplemental methods and supplemental notes.
(DOCX)
