# Supplementary figures and images for: Functional Dissection of Regulatory Models Using Gene Expression Data of Deletion Mutants
Source: PLoS Genet. 2013 Sep 5;9(9):e1003757. doi: 10.1371/journal.pgen.1003757 (PMC3764135; doi:10.1371/journal.pgen.1003757)

Figure S1

A

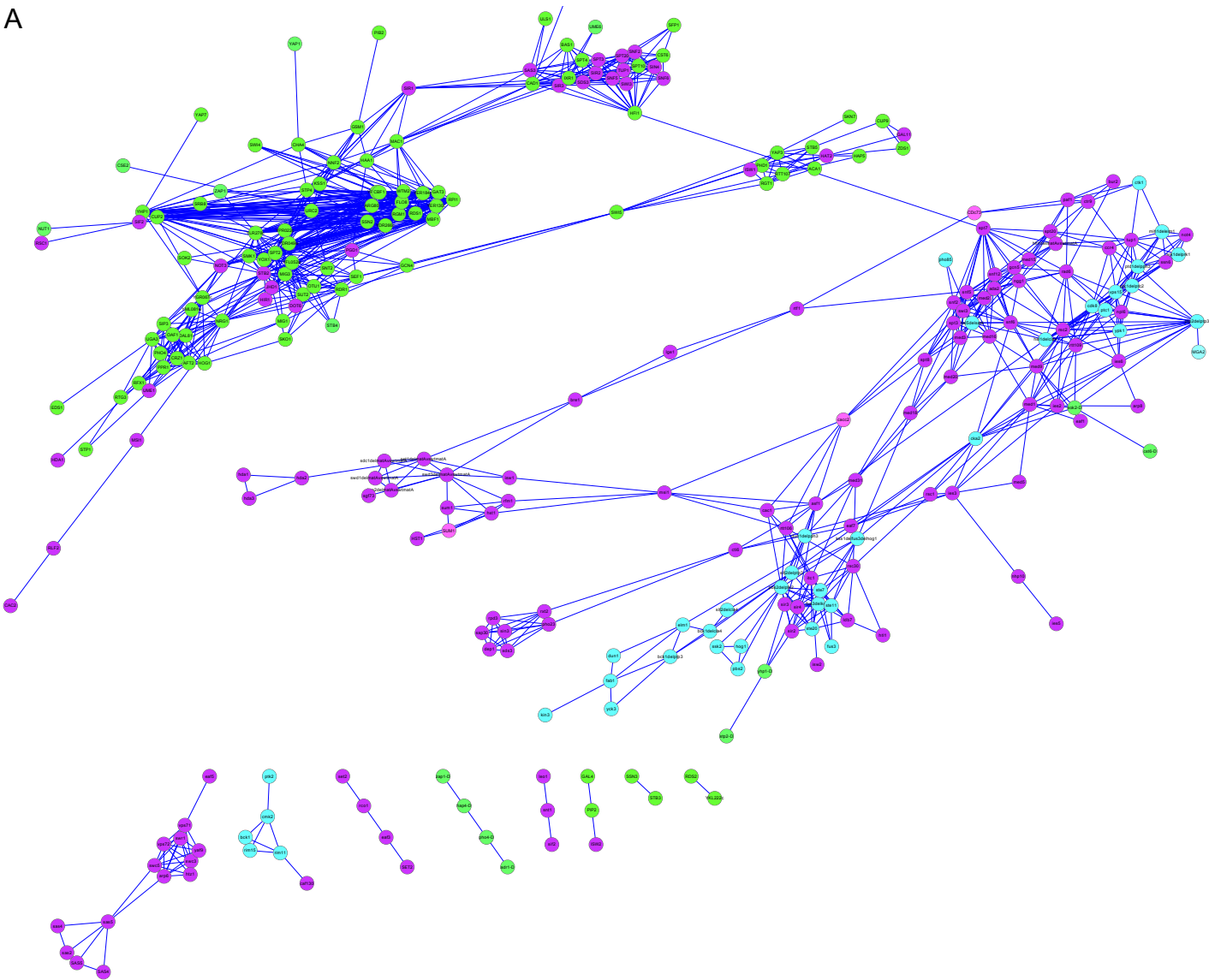

B

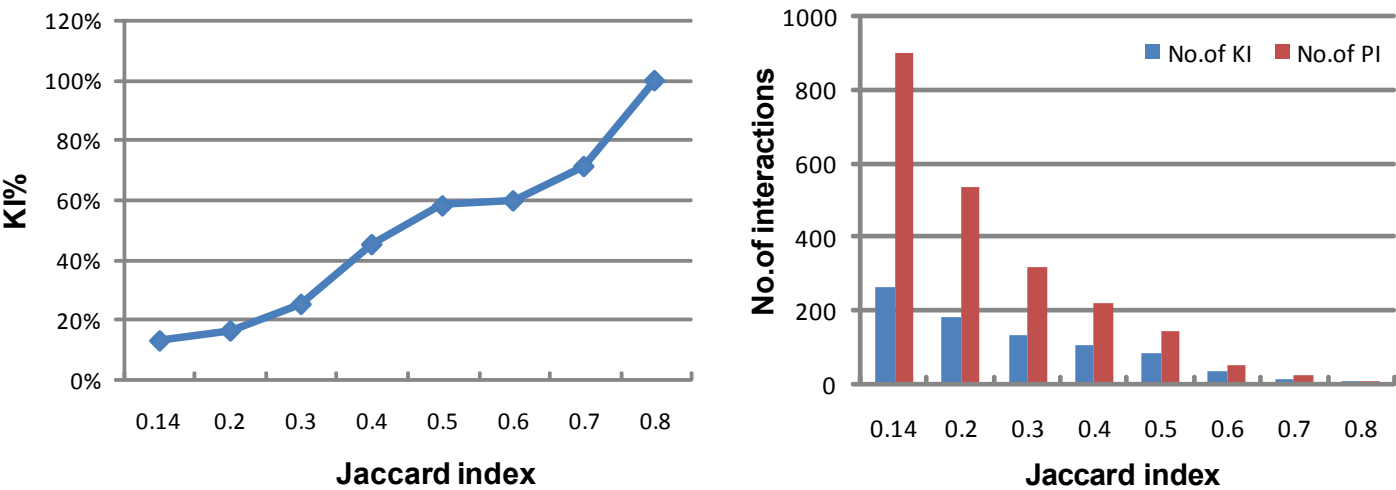

Supplement: Figure S1 — Jaccard's similarity between the target gene sets of regulators. (A) Dense pair-wise similarities between the gene expression profiles of deletion mutants. Edges connecting two nodes were drawn in the figure if the Jaccard similarity index between their targeting gene sets is higher than a specific threshold . A STF (Green nodes) is more likely to connect to another STF than to a GTF (Purple nodes). (B) Left panel: the similarity level of deletion mutant expression profiles (quantified by the thresholds of Jaccard's index used in the prediction) is correlated with the likelihood of being known interactions (protein-protein interactions and epistatic relationships downloaded from SGD). Right panel: The number of predicted and known interactions that passed the thresholds of Jaccard's similarity index. KI: known protein-protein interaction and epistatic relationships. PI: predicted protein-protein interaction and epistatic relationships. KI%: the percentage of known protein-protein interactions or epistatic relationships among gene pairs that passed the thresholds of Jaccard's similarity index. Purple nodes: chromatin machinery components (GTFs); Cyan nodes: single or double mutants of protein kinases, phosphatases; Green nodes: sequence-specific DNA binding transcription factors (STF). (PDF) [file pgen.1003757.s001.pdf]

Figure S2

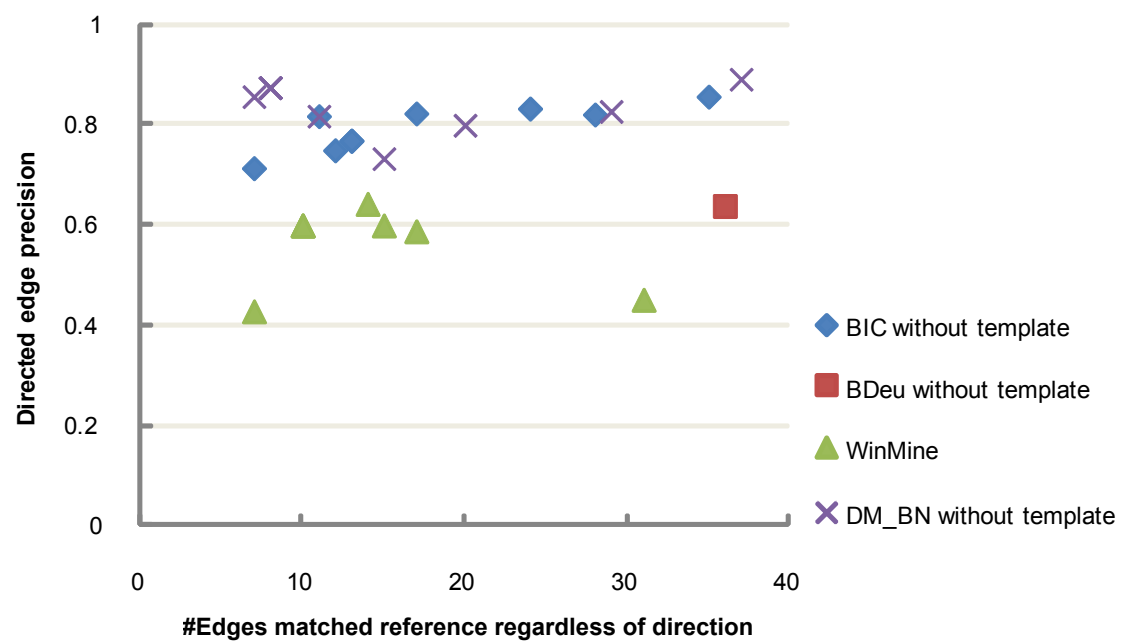

Supplement: Figure S2 — Precision of edge orientations for networks inferred by DM_BN, BIC, WinMine and BDeu at different parameters. BNs are predicted by four template-free BN learning algorithms (as indicated in the key) at various parameter settings. Using the regulator-DEGs identified in the deletion mutants experiments as reference, the x-axis represents the total number of predicted causal interactions in a BN (i.e., the directed edges in the corresponding PDAG) that overlap with the reference regardless of edge direction. The y-axis represents the precision of edge orientation (the percentage of predicted causal interactions with the same orientation as the reference). Tunable parameters include the kappa parameter in WinMine, the weight of the penalty term in BIC and the parameter in DM_BN. The BDeu metric does not have any tunable parameter and only predicts one network. Note that the recall of a BN prediction cannot be calculated based on these reference relationships, because simply linking regulators to DEGs identified from the deletion mutants experiments are too permissive to represent the direct regulator-target gene relationships and hence cannot be used as a gold standard. DEG: Differentially expressed genes. (PDF) [file pgen.1003757.s002.pdf]

Figure S3

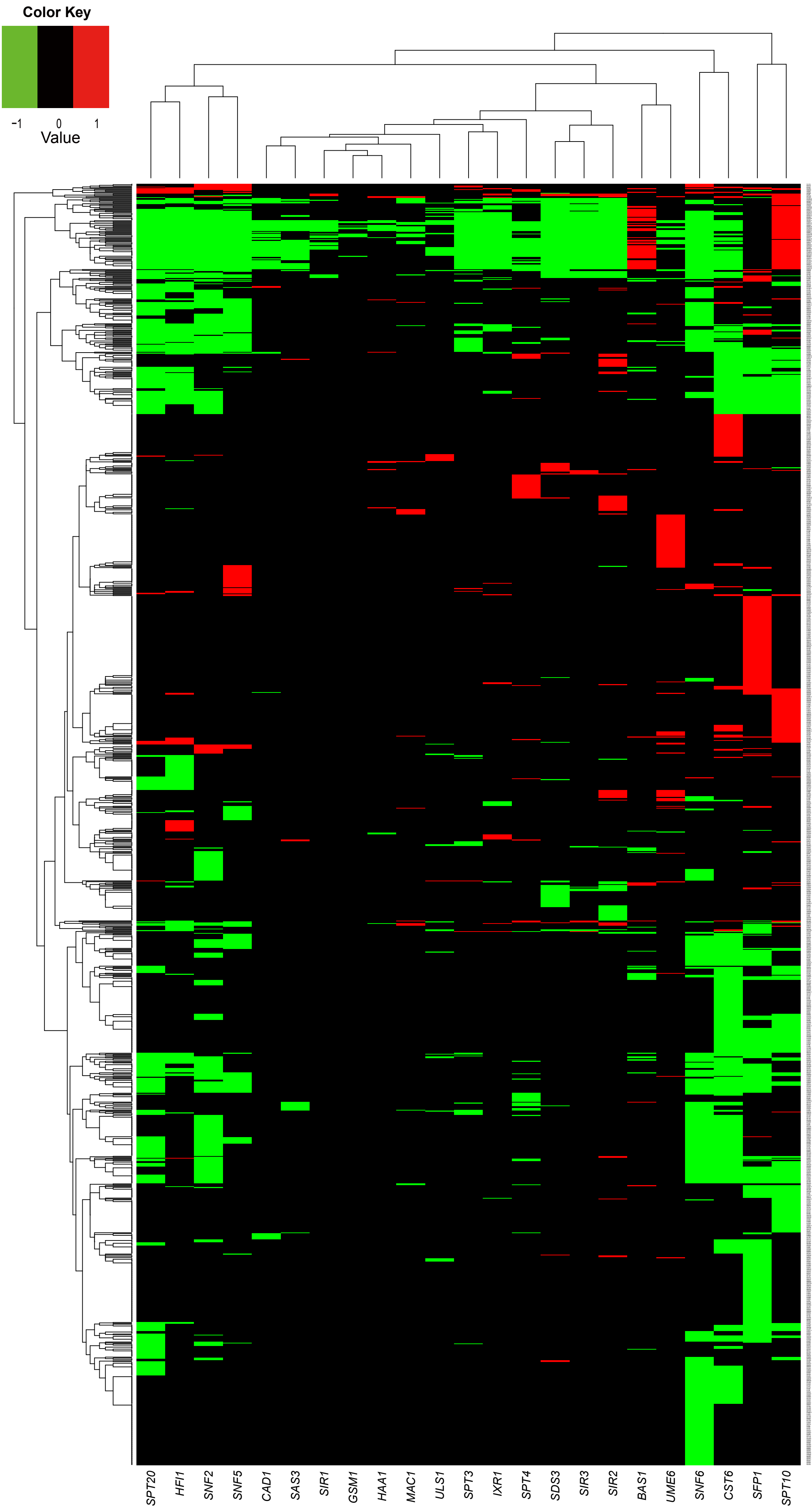

Supplement: Figure S3 — Clustering of the deletion mutant expression profiles of the STFs and GTFs shown in Figure 5C. (PDF) [file pgen.1003757.s003.pdf]
