# Supplementary material for: Functional Dissection of Regulatory Models Using Gene Expression Data of Deletion Mutants
Source: PLoS Genet. 2013 Sep 5;9(9):e1003757. doi: 10.1371/journal.pgen.1003757 (PMC3764135; doi:10.1371/journal.pgen.1003757)
Supplement: Table S3 — Function enriched among a regulator's target genes under rich medium growth conditions. (PDF) [file pgen.1003757.s006.pdf]

Table S3 Functional enrichment of regulators based on their target genes under rich medium growth conditions

Cellular process	Number of genes		Regulators involved in the cellular process	*P*-value
	In the cell process	mRNA expression significant changed		
[P]Amino acid metabolism	166	148	swi3,cbf1,mga2,taf14,sin4,,arg80,snf5,bas1,rsc2,snf2,snf6,rtg3,ric1,arg81,cdc73,sin3,ngg1,leu3,tup1,ume6,pho23,spt20,,aft1,cse2,cst6,sif2, stp1,eds1,srb2,pho2,gcn4,msn4, zap1,ada2,arp8,asf1,bre1,bre2,bur,caf40,ccr4,cdc73,chd1,cti6,ctr9,dls1,dpb4,eaf5,eaf7,gcn5,htz1,ies2, ies3,ies5,ies6,itc1,leo1,lge1,med1,med15,med16,med18,med2,med20, med3,med31,med5,med9,ngg1,nhp10,not3,not4,npl6,paf1,pho23,rad6,rpd3,rsc1,rsc2,rsc30,rtf1,rtg2, rtt106,rxt2,sap30,set1,sgf29,sif2,sin3,snf12,snf2,snf5,snf6,spt3,spt7,spt8, ssn6,swc3,swd3,swi3,vps71,yaf9,bck1delcla4,dun1delpph3, fab1,hal5delsat4,hsl1delcla4,pho85,ptc1,ptc1delpph3,sit4,sky1,snf1,snf1delrim11,vps15,yck3,ypk1,yvh1	≤0.01
[P]Carbohydrate metabolism	212	195	cbf1,flo8,mga2,,sin4,gal80,arg80,rgm1,wtm2,ssn2,yer184c,snf6,ric1,mbf1,gcr2,sin3,yer130c,tup1,rds1,cst6,gat3,qis1,rox1,sok2,ada2,bur2,cti6,dls1,dpb4,hda1,hda2,hda3, hfi1,htz1,ies2,ies3,ies6,isw1,med1,med15,med2,med3,med9,ngg1,not3,npl6,rsc1,rsc2,rtg2,rtt109,spt20,spt7,ssn6,sus1,swc5,tup1,yaf9,arkdelprk1,cdk8,cka2,dun1,elm1, hsl1delcla4,ire1,mih1delelm1,ptc1,ptc1delpph3,ptc1delptc2,ptp2delptp3,sit4,vps15	≤0.01
[P]Signal transduction	71	52	dig1,taf14,hat2,cdc73,phd1,esc2,pho23,gln3,rgt1,sds3,aca1,gis2,msn4,skn7,,ste12,stp2,yhp1,ada2,cti6, gcn5,htl1.med16,nph6,sgf29,sir2,sir3,sir4,spt20,spt7,sus1fab1, fus3,fus3delkss1,hog1,kin3,kss1delfus3delhog1,pbs2,pbs2delptk2,ssk2,ste11,ste20,ste7	≤0.01
[P]mating conjugation	106	78	dig1,sir3,cdc73,,phd1,sir2,rgt1,sds3,skn7,ste12,stp2,,yhp1,chd1,cti6,dep1,dpb4,gcn5,htl1, isw2,itc1,ldb7,not4,pho23,rpd3,rxt2,sgf29,sir2,sir3,sir4,snf6,spt20,spt7,ssn6,sus1, tup1,rsc30,rxt2,sap30,sin3,fus3,fus3delkss1,kss1delfus3delhog1,pbs2,pbs2delptk2,ste11,ste20,ste7	≤0.05
cell wall organization or biogenesis	210	165	cbf1,set2,hst1,ric1,hog1,rme1,sum1,yhp1,swi4,rco1,crz1,rgm1,sps18,bre1,cac1,chd1,hst1,htl1,lge1,med15,med2,med3,npl6,rfm1,sdc1,set1,sgf73,snt1,sum1, rsc2,asf1, ark1delprk1,bck1,cdk8,fab1,hsl1delcla4,kin3,mih1delelm1,ptc1,ptc1delpph3,ptc1delptc2,ptp2delptp3,sit4,vps15,yck3,ypk1,slt2delptp3.pho85,fus3	≤0.05
[P]Energy metabolism	139	112	swi3,sin4,snf6,gal11,pgd1,gcr2,ngg1,pho23,stp1,srb2,zap1,bre1,ccr4,dls1,dpb4,eaf1,hfi1,ies5,lge1,med15,med16,med2,med3,med9,ngg1,nhp10,npl6, rtf1,rtg2,rtt109,sgf29,snf12,snf2,snf6,spt20,spt3,spt8,ssn6,swc3,swi3,swr1,wtypdvswt,yaf9,ark1delprk1,hal5delsat4,sit4,sky1,snf1delrim11	≤0.01

[P]Nucleotide Metabolism	115	83	rfx1,taf14,bas1,snf2,snf6,ppr1,gln3,cst6,pho2,sfp1,rfx1,ada2,eaf5,ngg1,snt1,swc3,vps72,yaf9,cka1,oca1,siw14,sky1,snf1delrim11,tup1.sit4,yvh1	≤0.05
[P]Lipid Metabolism	112	92	mga2,opi1,ric1,spt20,aft1,stp3,cdc73,ctr9,dls1,hfi1,ies2,ies6,med15,med2,sgf73,snf12,snf2,snf5,snf6,spt20,spt7,ssn6,tup1,ark1delprk1,cdk8,ire1,snf5,bur2,swi3	≤0.05
Protein folding	88	70	sin4,snf6,stb1,aft1,cst6,nut1,skn7,bur2,caf130,eaf7,ies2,nhp10 ,paf1,snt1,chk1,ire1	≤0.05
Ribosome biogenesis, organization	230	169	snf2,snf6,arg81,spt10,spt20,hal9,cst6,ime1,sfp1,gat4,tos8,rtt109,ark1delprk1,yrr1,rfx1,hfi1,isw2	≤0.05
Mitochondrion relations	337	219	srb8,mal33,stb1,dal82,nut1,thi2,adr1,ecm22,hac1,mbp1,rgm1,sip4,ccr4,hac1	≤0.1
DNA replic ation, repair	316	188	jhd1,nut1.gis2,cat8,gis2,mbp1,sut1,tos8	≤0.1
response to osmotic stress	77	54	hog1,pho23,hal9,msn4 ,spt2,hog1,pbs2,pbs2delptk2,ptp2delptp3,ptc1,ste11	≤0.05
Sporulation	131	59	swi6,hst1,sum1,ino2,sps18,bre1,cac1,cac2,cti6,hst1,isw1,med15,med2,med3,msi1,not3,rfm1,rtf1,sdc1,set1,sgf73,sif2,sum1,swd1,swd3,tup1,bck1delptp3,mck1,slt2delptp3	≤0.05
vesicle organization	77	44	mig3,cup2,rco1,not4,gat3,arp6,htz1,swc3,swc5,swr1,vps71,vps72,yaf9	≤0.1
vitamin metabolic process	47	34	cac2,set2,sas4,ric1,gzf3,sko1,spt10,sas5,rlf2,hfi1,mot3,tos8,ada2,bre2,caf40,cti6,dep1,dls1,eaf3,eaf5,eaf7,hfi1,htz1,ldb7,med15,not4,npl6,pho23,rsc1,rxt2,sap30,sas2,sas4, sas5,sds3,set1,sgf73,sin3,snf12,snf2,snt1,spt20,spt3,spt7,spt8,ssn6,swd3,cka1,ksp1,mih1delelm1,sit4,yvh1	≤0.05
pseudohyphal growth	68	49	sip3,sok2,gzf3,phd1,hda1,not3,ste12,ykl222C,ste12,med16,med2,med3,snf12,elm1,ste11,ste20,ste7,yck3	≤0.05

P: genes involved in the cellular process were downloaded from the KEGG pathway database, others were downloaded from the GO terms database in SGD
