# Supplementary material for: Functional Dissection of Regulatory Models Using Gene Expression Data of Deletion Mutants
Source: PLoS Genet. 2013 Sep 5;9(9):e1003757. doi: 10.1371/journal.pgen.1003757 (PMC3764135; doi:10.1371/journal.pgen.1003757)
Supplement: Table S4 — Evaluation of the WinMine and DM_BN algorithm based on known knowledge of chromatin modification complex. For a fair comparison, parameters for the two algorithms were set to in WinMine and in DM_BN, so that the networks inferred by different methods have roughly the same number of edges. (DOCX) [file pgen.1003757.s007.docx]

**Table S4** **Evaluation of the WinMine and DM_BN algorithm based on known knowledge of chromatin modification complex.**

| ***Method*** | ***No. of edges**** | ***Recall*** | ***Precision*** | ***Fisher test*** |
| --- | --- | --- | --- | --- |
|  |  |  |  | ***P-value*** |
| WinMine | 119 | 0.04639 | 0.7731 | <2.2e-16 |
| DM_BN | 115 | 0.04942 | 0.85217 | <2.2e-16 |

*: the number of edges in chromatin modification complex predicted by WinMine and DM_BN
