# Supplementary material for: Functional Dissection of Regulatory Models Using Gene Expression Data of Deletion Mutants
Source: PLoS Genet. 2013 Sep 5;9(9):e1003757. doi: 10.1371/journal.pgen.1003757 (PMC3764135; doi:10.1371/journal.pgen.1003757)
Supplement: Table S5 — Similarity between the target gene sets of subunits of the SWR1 complex and Htz1, which are involved in vesicle organization. See Figure 3 for their predicted relationships by DM_BN. (DOCX) [file pgen.1003757.s008.docx]

**Table S5.** **The similarity between the target gene sets of subunits of the SWR1 complex and Htz1.**

| **Reg1** | **Reg2** | **#Common targets of**  **Reg1, Reg2** | | **#Targets of Reg1** | **#Targets of Reg2** | **JI(Reg1,Reg2)** |
| --- | --- | --- | --- | --- | --- | --- |
| yaf9 | htz1 | | 24 | 42 | 68 | 0.27906 |
| arp6 | htz1 | | 18 | 24 | 68 | 0.24324 |
| swc5 | htz1 | | 24 | 26 | 68 | 0.34285 |
| vps72 | htz1 | | 17 | 21 | 68 | 0.23611 |
| swr1 | htz1 | | 21 | 33 | 68 | 0.2625 |

Reg1: regulator1.

Reg2: regulator2.

*#*: number of the targets genes in deletion mutant of regulator 1, regulator 2 and in both of them

*JI*: Jaccard similarity index between the target gene sets of the two regulators.
