# Supplementary material for: Functional Dissection of Regulatory Models Using Gene Expression Data of Deletion Mutants
Source: PLoS Genet. 2013 Sep 5;9(9):e1003757. doi: 10.1371/journal.pgen.1003757 (PMC3764135; doi:10.1371/journal.pgen.1003757)
Supplement: Table S11 — Evaluating the causality predicting performance of WinMine, BIC and DM_BN with/without the network template using known knowledge of the MAPK signaling pathways. The relationships were predicted by WinMine under the parameter setting , by BIC under the penalty term's weight 2.0 and by DM_BN under . (PDF) [file pgen.1003757.s014.pdf]

Table S11 Evaluating the causality predicting performance of BN learning algorithms using known knowledge of the MAPK signaling pathways.

Algorithm	Osmolyte synthesis	Mating, Filamentation	Cell wall remodeling	Precision EM*	Precision CD#	Recall EM*	Recall CD#	Fisher test P-EM*	Fisher test P-CD#
MAPK signaling pathways predicted by WinMine	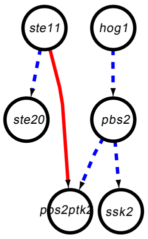	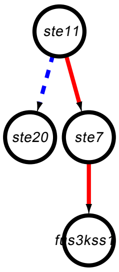	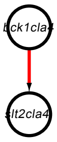	0.4444	0.4167	0.3333	0.2174	0.1109	0.7012
MAPK signaling pathways predicted by BIC without template	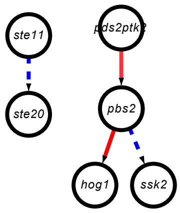	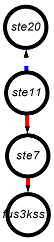	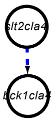	0.5000	0.5455	0.3333	0.2609	0.0715	0.3306
MAPK signaling pathways predicted by BIC with template	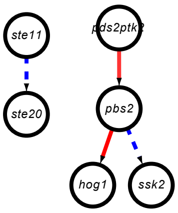	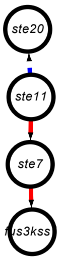	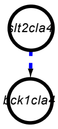	0.5000	0.5455	0.3333	0.2609	0.0715	0.3306
MAPK signaling pathways predicted by DM_BN without template	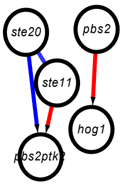	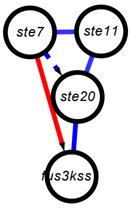	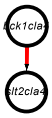	0.6667	0.8333	0.3333	0.2174	0.0239	0.0529
MAPK signaling pathways predicted by DM_BN with template	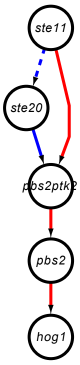	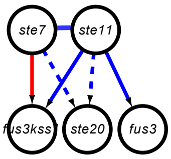	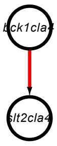	0.4545	0.8125	0.4167	0.5652	0.0617	0.00044

Predicted edges that have the same orientations as the known interactions in MAPK signaling pathways are shown as red solid lines; Edges that have consistent directionalities (but through indirect connections in the pathways) are shown as blue solid lines; Edges without any known MAPK pathway knowledge support are shown as blue dashed lines.

EM*: Precision, Recall and Fisher test results based on the exactly matched edges shown by the red solid lines.

CD#: Precision, Recall and Fisher test results based on the edges with correct directions shown by the solid lines.
