# Supplementary material for: Functional Dissection of Regulatory Models Using Gene Expression Data of Deletion Mutants
Source: PLoS Genet. 2013 Sep 5;9(9):e1003757. doi: 10.1371/journal.pgen.1003757 (PMC3764135; doi:10.1371/journal.pgen.1003757)
Supplement: Table S12 — Similarity between target gene sets of subunits in the SIR complex and kinases in the STE-mediated MAPK signaling pathways. In this table, we list the Jaccard index similarities between regulators involved in mating and filamentous growth processes. See Figure 3 for more information for their predicted relationships. (DOCX) [file pgen.1003757.s015.docx]

**Table S12 The Jaccard similarity between target gene sets of subunits in the SIR complex and kinases in the STE MAPK pathway.**

| **JI(****SIR)** | **JI (****STE)** | *JI*(STE, SIR) |
| --- | --- | --- |
| *JI*(sir2, sir4)=0.75 | *JI*(ste20=-1, ^a^fk=-1; ste7)=0.43 | *JI*(ste7, sir2)=0.36 |
| *JI*(sir3, sir4)=0.84 | *JI*(ste20=-1, ^a^fk=-1; ste11)=0.53 | *JI*(ste11, sir4)=0.35 |
| *JI*(sir2, sir3)=0.75 | *JI*(ste20, ^a^fk)=0.44 | *JI*(ste7∩ste11, sir3)=0.32 |

SIR: SIR Complex

STE: STE mediated MAPK signal transduction pathway.

^a^fk: fus3;kss1, *i.e.*, double mutant of fus3 and kss1.

JI: Jaccard index
