# Supplementary material for: Functional Dissection of Regulatory Models Using Gene Expression Data of Deletion Mutants
Source: PLoS Genet. 2013 Sep 5;9(9):e1003757. doi: 10.1371/journal.pgen.1003757 (PMC3764135; doi:10.1371/journal.pgen.1003757)
Supplement: Text S1 — Supplemental methods and supplemental notes. (DOCX) [file pgen.1003757.s016.docx]

**Text S1 Supplemental Methods and Supplemental Notes**

**Supplemental Methods**

**Microarray data normalization**

For data sets GSE25644 and GSE25909, microarray data normalization was performed based on mean intensity values using print-tip LOESS as described in Yang et al [[1](#_ENREF_1)] (implemented in the marray R package version 1.20.0, we use no background subtraction and a window span of 0.4 [[2](#_ENREF_2),[3](#_ENREF_3)]). For dataset GSE4564, the relative expression level of each gene in the mutant strain to the wild type was calculated, normalizing for growth and batch effects, as described in [[4](#_ENREF_4),[5](#_ENREF_5)]. Normalization of data set GSE2324 was already carried out in the Rosetta Resolver database (mean $\log_{2}$ratio was normalized to zero for the entire array), as described in [[6](#_ENREF_6),[7](#_ENREF_7)].

**Statistical analysis of expression profiles**

Overall, for different microarrays, we used the same normalization and significance testing methods as described in the original papers and the according *P-*value cutoffs therein. *P-*values of gene expression alternation in dataset GSE25644 and GSE25909 were obtained from the *limma* R package version 2.12.0 [[8](#_ENREF_8)], after Benjamini-Hochberg FDR correction. Gene expression changes were considered significant when fold change $FC>1.7$ and $P-\text{value}\leq0.05$ as described in [[2](#_ENREF_2),[3](#_ENREF_3)]. In dataset GSE2324, *P-*values were calculated after Bonferroni correction. Gene expression changes were considered significant when fold change $FC>2$ and $p-\text{value}\leq0.01$ as described in [[7](#_ENREF_7)]. In data set GSE4564, the value $\log_{10}(\frac{G}{R})$ (G, R indicate the intensity of green and red signals) was used directly to compute a mean ratio value by the minimum-variance weighted average method [[9](#_ENREF_9),[10](#_ENREF_10)]. The error of the mean ratio was then used to estimate and derive an *X* score which was further used to represent the confidence level of the averaged measurement [[10](#_ENREF_10)]. The *X* score was then used to compute the mean significance ratios (which can be used to estimate the corresponding *P*-values). Genes were considered significantly changed when $P-\text{value}\leq0.001$as described in [[5](#_ENREF_5)].

To avoid **using** non-informative training data, we removed from train cases those genes that were not differentially expressed in at least one deletion mutant experiment. Besides, we found that the average expression levels of 10 genes have a significant change between wild type (WT) yeast grown in the YPD and SC mediums, which reflects the background noise. Therefore, we excluded the regulatory genes whose deletion mutant strains result in less than 10 differentially expressed genes (DEGs) when compared to the wild type to be nodes in the inferred networks, since their impact to the transcriptome are less than or equal to a background effect due to routine culture medium differences.

**Supplemental Notes**

1. **A brief introduction of the network-inference methods tested in this work**

ARACNE [[11](#_ENREF_11)] is a method for inferring regulatory networks using the mutual information criterion; the Disruption Network approach [[12](#_ENREF_12)] builds a regulatory network by linking genetically perturbed TFs with differentially expressed genes and the Jaccard index (*JI*) approach links node pairs whose *JI* similarity passed a pre-set cutoff to construct regulatory networks; The WinMine Toolkit [[13](#_ENREF_13)] is a popular BN inference software developed by Microsoft Research; The Bayesian Information Criterion (BIC) score is a general criterion for selecting statistical models [[14](#_ENREF_14)] and it has been used to score different Bayesian network structures [[15](#_ENREF_15)]. The Bayesian Dirichlet equivalence uniform (BDeu) score [[16](#_ENREF_16)] is another widely used criterion to score Bayesian network structures. It has a tunable parameter, the equivalent sample size (ESS), which could affect the performance of this scoring approach. In our implementation, we employed an iterative approach to set this parameter to its optimal value [[17](#_ENREF_17)].

1. **Comparison of the performance of DM_BN with other BN learning algorithms in predicting causal relationships**

We compared the performance of different BN learning algorithms in *de novo* predicting causal relationships without using *a priori* regulator-DEG information. In this test, we compared the directed (compelled) edges in the PDAG (inferred by these algorithms without using template) to the regulator-DEG relationships (the default network template used for DM_BN), as although encoded in the same datasets, the transcriptome similarities between deletion mutant strains used by template-free BN learning is an independent dimension of information from the explicit information of individual regulator-target relationships (since the identities (names) of the DEGs in each deletion-mutant experiment are kept blind to the BN algorithms, they could not infer explicit regulator-target relationships directly from data). Furthermore, the relatively large amounts of such relationships made it possible to compare the *de novo* edge orientation performance of different BN learning algorithms quantitatively and statistically. To measure the precision of edge orientation, we computed the numbers of matched edges considering or not considering edge orientation. Then, the precision of orientation is defined as the ratio of the two numbers and the Binomial test is used to estimate the statistical significance of the consistency of edge orientations with the regulator-DEG relationships.

It is clear that among the four tested BN learning algorithms, the DM_BN algorithm and the BIC scoring approach [[14](#_ENREF_14),[15](#_ENREF_15)] can generally predict correct causal relationships *de novo* (See Supplemental Note 3 for more discussions), while the precision of edge orientations predicted by the BDeu scoring method [[16](#_ENREF_16)] or the WinMine toolkit [[13](#_ENREF_13)] is only marginally better than random coin tossing (Figure S2). Clearly, they are not the top runners for the deletion mutant datasets, although they might be still useful at predicting causal relationships in other application scenarios.

**3. High precision prediction of causal relationship by the DM_BN algorithm**

Upon a closer look at Figure S2, we can see the precision of edge orientations predicted by the DM_BN algorithm is higher than 80% for all but one parameter setting. Moreover, it is interesting to see that among the networks predicted by DM_BN, the network with the largest number of edges has the highest precision of edge orientation (89.19%, 33 over 37 overlapped edges have correct directionality). As a result, a good strategy to call the DM_BN algorithm might be first executing it at the smallest value of the $w$ parameter (Methods) to obtain a PDAG network with the largest number of edges and then use it to orient the corresponding edges of networks inferred at other parameter settings. In this sense, the DM_BN algorithm has the best performance of predicting edge causality among all the tested BN algorithms.

**Supplemental References**

1. Yang YH, Dudoit S, Luu P, Lin DM, Peng V, et al. (2002) Normalization for cDNA microarray data: a robust composite method addressing single and multiple slide systematic variation. Nucleic Acids Research 30.

2. Holsteget FCP, Lenstra TL, Benschop JJ, Kim T, Schulze JM, et al. (2011) The Specificity and Topology of Chromatin Interaction Pathways in Yeast. Molecular Cell 42: 536-549.

3. Holstege FCP, van Wageningen S, Kemmeren P, Lijnzaad P, Margaritis T, et al. (2010) Functional Overlap and Regulatory Links Shape Genetic Interactions between Signaling Pathways. Cell 143: 991-1004.

4. Eisen MB, Brown PO (1999) DNA arrays for analysis of gene expression. Cdna Preparation and Characterization 303: 179-205.

5. Iyer VR, Hu ZZ, Killion PJ (2007) Genetic reconstruction of a functional transcriptional regulatory network. Nature Genetics 39: 683-687.

6. Roberts CJ, Nelson B, Marton MJ, Stoughton R, Meyer MR, et al. (2000) Signaling and circuitry of multiple MAPK pathways revealed by a matrix of global gene expression profiles. Science 287: 873-880.

7. Dion MF, Altschuler SJ, Wu LF, Rando OJ (2005) Genomic characterization reveals a simple histone H4 acetylation code. Proceedings of the National Academy of Sciences of the United States of America 102: 5501-5506.

8. Smyth GK, Michaud J, Scott HS (2005) Use of within-array replicate spots for assessing differential expression in microarray experiments. Bioinformatics 21: 2067-2075.

9. Harbison CT, Gordon DB, Lee TI, Rinaldi NJ, Macisaac KD, et al. (2004) Transcriptional regulatory code of a eukaryotic genome. Nature 431: 99-104.

10. Hughes TR, Marton MJ, Jones AR, Roberts CJ, Stoughton R, et al. (2000) Functional discovery via a compendium of expression profiles. Cell 102: 109-126.

11. Margolin AA, Nemenman I, Basso K, Wiggins C, Stolovitzky G, et al. (2006) ARACNE: an algorithm for the reconstruction of gene regulatory networks in a mammalian cellular context. BMC Bioinformatics 7 Suppl 1: S7.

12. Rung J, Schlitt T, Brazma A, Freivalds K, Vilo J (2002) Building and analysing genome-wide gene disruption networks. Bioinformatics 18 Suppl 2: S202-210.

13. Chickering DM (2002) The WinMine Toolkit. Microsoft.

14. Schwarz G (1978) Estimating the Dimension of a Model. The Annals of Statistics 6: 461-464.

15. Heckerman D (1999) A Tutorial on Learning with Bayesian Networks. In: Jordan M, editor. Learning in Graphical Models. Cambridge, MA: MIT Press.

16. Heckerman D, Geiger D, Chickering DM (1995) Learning Bayesian Networks: The Combination of Knowledge and Statistical Data. Machine Learning 20: 197-243.

17. Steck H (2008) Learning the Bayesian Network Structure: Dirichlet Prior vs Data. In: McAllester DA, Myllymaki P, editors. UAI 2008, Proceedings of the 24th Conference in Uncertainty in Artificial Intelligence. Helsinki, Finland: AUAI Press. pp. 511-518.
